# The genome sequence of the biocontrol fungus *Metarhizium anisopliae* and comparative genomics of *Metarhizium* species

**DOI:** 10.1186/1471-2164-15-660

**Published:** 2014-08-07

**Authors:** Julie A Pattemore, James K Hane, Angela H Williams, Bree AL Wilson, Ben J Stodart, Gavin J Ash

**Affiliations:** Graham Centre for Agricultural Innovation, School of Agricultural and Wine Sciences, Charles Sturt University, Locked Bag 588, Wagga Wagga, 2650 NSW Australia; Black Box Bioinformatics, Perth, WA Australia; Centre for Crop and Disease Management, Department of Environment and Agriculture, Curtin University, Perth, WA Australia

**Keywords:** *Metarhizium anisopliae*, Genome, Pathogenicity, Motifs, Mating-type, Comparative, Genomics

## Abstract

**Background:**

*Metarhizium anisopliae* is an important fungal biocontrol agent of insect pests of agricultural crops. Genomics can aid the successful commercialization of biopesticides by identification of key genes differentiating closely related species, selection of virulent microbial isolates which are amenable to industrial scale production and formulation and through the reduction of phenotypic variability. The genome of *Metarhizium* isolate ARSEF23 was recently published as a model for *M. anisopliae*, however phylogenetic analysis has since re-classified this isolate as *M. robertsii*. We present a new annotated genome sequence of *M. anisopliae* (isolate Ma69) and whole genome comparison to *M. robertsii* (ARSEF23) and *M. acridum* (CQMa 102).

**Results:**

Whole genome analysis of *M. anisopliae* indicates significant macrosynteny with *M. robertsii* but with some large genomic inversions. In comparison to *M. acridum*, the genome of *M. anisopliae* shares lower sequence homology. While alignments overall are co-linear, the genome of *M. acridum* is not contiguous enough to conclusively observe macrosynteny. Mating type gene analysis revealed both *MAT1-1* and *MAT1-2* genes present in *M. anisopliae* suggesting putative homothallism, despite having no known teleomorph, in contrast with the putatively heterothallic *M. acridum* isolate CQMa 102 (*MAT1-2)* and *M. robertsii* isolate ARSEF23 (altered *MAT1-1*). Repetitive DNA and RIP analysis revealed *M. acridum* to have twice the repetitive content of the other two species and *M. anisopliae* to be five times more RIP affected than *M. robertsii.* We also present an initial bioinformatic survey of candidate pathogenicity genes in *M. anisopliae*.

**Conclusions:**

The annotated genome of *M. anisopliae* is an important resource for the identification of virulence genes specific to *M. anisopliae* and development of species- and strain- specific assays. New insight into the possibility of homothallism and RIP affectedness has important implications for the development of *M. anisopliae* as a biopesticide as it may indicate the potential for greater inherent diversity in this species than the other species. This could present opportunities to select isolates with unique combinations of pathogenicity factors, or it may point to instability in the species, a negative attribute in a biopesticide.

**Electronic supplementary material:**

The online version of this article (doi:10.1186/1471-2164-15-660) contains supplementary material, which is available to authorized users.

## Background

**Figure 1:**
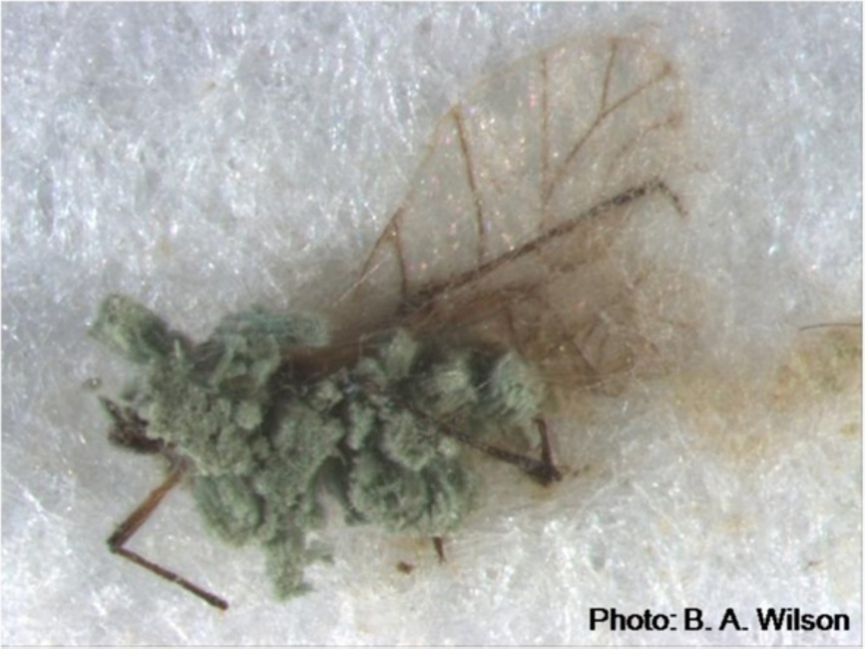
**Green conidia of** ***Metarhizium anisopliae*** **isolate Ma69 erupting from the cadaver of a rose-grain aphid (** ***Metopolophium dirhodum*** **).**

*Metarhizium anisopliae* is a globally distributed, entomopathogenic fungus that infects many important crop pests including aphids, scarabaeoid beetle larvae and western flower thrips [1–4] (Figure 1). The species was one of the first to be investigated for its use as a biological control agent and advances in the understanding of its biology and ecology have led to improved biocontrol applications [5]. *M. anisopliae* is regarded as asexual as no teleomorph has been observed [6]. In cases such as these, phylogenetic species boundaries are often used to taxonomically characterize anamorphic fungi [7] and *M. anisopliae* has been well characterized in this regard [5, 6, 8, 9]. Analysis of mating type loci (idiomorphs) however, can enhance our understanding of the genetic mechanisms behind sexual or asexual lifestyles and the potential pathways of genetic exchange.

**Figure 2:**
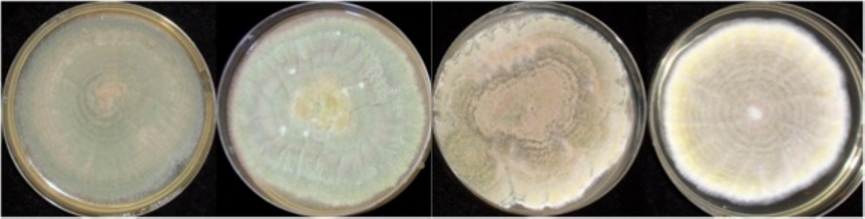
**Examples of cultural variability found among single spored isolates from the parent culture of** ***M. anisopliae*** **BRIP 53293, grown on SDAY plates of identical composition under identical temperature regimes.** The main morphologies observed are (from left to right) one highly sporulating olive green culture and low sporulating tri-colour cultures.

The *Metarhizium* species complex is diverse, including generalist species with a broad host range and specialist species with narrow host ranges. Furthermore, individual isolates can also exhibit a range of cultural variability. In our laboratory, variability of cultures is minimized through the use of single spore isolates and mother cultures from long term storage. Despite best practice, significant variability arises in the colour and amount of sporulation from replicates of the same single spore isolates (BRIP 53293) cultured on SDAY plates of identical composition and grown under identical temperature regimes (Figure 2). Two main morphologies have been observed: 1) highly sporulating olive green cultures and 2) low sporulating tri-colour cultures, that is orange, pale green and olive green. In addition to these morphologies, some cultures also exhibit more abundant fluffy mycelial growth while others tend to sector, a sign of aging [10]. Culture degeneration has been shown to affect the stability of enzyme production (e.g. cuticle degrading enzymes) and secondary metabolite production (e.g. destruxins) and results in extensive downstream gene regulation [10, 11].

While possible mechanisms responsible for sectoring may include changes due to physiological and environmental adaptations or differences in transposable elements [11–13], our observations of cultural phenotype variation under laboratory conditions are not well understood. Phenotypic variability which affects sporulation in the laboratory could also affect the multiplication of blastospores in the host haemocoel, isolate pathogenicity, as well as commercial conidial production, which has important implications for maintenance, preservation and the selection of isolates for commercialization [11, 14]. The existence of unique divergent gene sets and expansion of gene families within *M. anisopliae* may provide an important genetic resource for the continued development of this important entomopathogen as a biopesticide.

The recent publication of reference genomes and comparative genomics of the generalist *M. anisopliae* and the locust-specific pathogen *M. acridum* has further enhanced our understanding of the biology of these entomopathogenic fungi and the molecular basis of host-specificity [5, 13]. However the isolate sequenced as the reference strain for *M. anisopliae* (ARSEF23) was subsequently re-classified to *M. robertsii* [13], a phylogenetically distinct species within the *Metarhizium* PARB clade (*M. pingshaense*, *M. anisopliae, M. robertsii* and *M. brunneum*) [5].

In light of the well-supported divergence of ARSEF23 from the main *M. anisopliae* clade [5, 9] and re-classification to *M. robertsii*, the primary aim of this study was to assemble an accurate, annotated, draft reference genome for *M. anisopliae* using our isolate Ma69. We used a comparative genomics approach, not to repeat the excellent work of the previous publication [13], but to complement it, add to the body of knowledge and establish new genomic resources for the *Metarhizium* genus. In this study, we compare the genomes of *M. anisopliae, M. robertsii* and *M. acridum* to identify the key suite of genes which differentiate *M. anisopliae* from the other two species. We used a multi-faceted bioinformatics approach to identify genes that were divergent between the three *Metarhizium* species and to assign putative functions to them where possible. We identify a suite of effector-like genes that are predicted to be specific to *M. anisopliae,* significant differences in the repetitive DNA complements, repeat-induced point mutations and mating type gene composition between the three species and discuss the implications of these findings.

## Results

### Phylogenetic validation, genome sequencing and assembly of *M. anisopliae*isolate Ma69

The rDNA internal transcribed spacer (ITS) region of *M. anisopliae* isolate Ma69 was sequenced and analyzed by BLASTN to confirm its identity [10]. One hundred hits to *M. anisopliae* ITS sequences with an e-value of 0.00 were obtained, confirming isolate Ma69 as *M. anisopliae* (Additional file 1. Top ten hits only).

The genome of isolate Ma69 was then shotgun-sequenced by generating two Illumina libraries, a 100 bp paired-end library and a 3 kb mate-paired library, comprising 5.61 Gb and 9.29 Gb of raw data respectively. This corresponded to approximately 380X coverage of the final genome assembly. A total of 142.9 million reads (96.02%) were retained for assembly after quality-control filters were applied for the removal of adapter sequence and regions of low base-call quality or low sequence complexity. Initial *de novo* assembly using only paired-end reads produced an assembly containing 1,567 scaffolds with an N50 of 138 and an N50 length of 85,355 bp. Contigs were scaffolded with the 3 kb mate-pair library, resulting in 577 scaffolds with an N50 of 11 and an N50 length of 1,243,138 bp. The genome assembly statistics and general features were tabled along with those of *M. robertsii* and *M. acridum* [13] (Table 1). The whole genome shotgun project of the organism *M. anisopliae*, isolate BRIP 53293 EFD69 SSC31 (Ma69), was deposited at DDBJ/EMBL-Bank/GenBank under the accession APNB00000000.

**Table 1:** **Summary of genome and gene content for** ***Metarhizium*** **isolates,** ***M. anisopliae*** **Ma69,** ***M. robertsii*** **ARSEF23 and** ***M. acridum*** **CQMa102**

Features	***M. anisopliae***Ma69	***M. robertsii***ARSEF23	***M. acridum***CQMa102
Genome assembly size (Mb)	38.6	39.04	38.05
Genome assembly coverage	380x	100x	107x
Genome assembly N50	11	7	36
Genome assembly N50 length	1,243,138	1,958,674	329,480
Total scaffolds in genome assembly	577	176	241
G + C content (%)	51.49	51.49	49.91
Protein-coding genes	11415	10582	9849
Average protein length (aa)	486	506	493
Gene density (genes per Mbp)	295.7	271.1	258.8
Average exons per gene	2.8	2.8	2.7
Predicted secreted proteins	1413	1865	1490
tRNA genes	132	141	122
Repetitive DNA (%) - *de novo* repeats	2.4	2.12	4.42
Repetitive DNA (%) - RepBase (taxon: “fungi”)	0.98	1.05	1.21
Overall RIP dominance (CpA ← → TpA)	1.0	0.22	1.14
Overall TA/AT (repetitive DNA)	1.02	1.02	1.31
Overall TA/AT (non-repetitive DNA)	0.7	0.69	0.77

### Whole-genome synteny comparisons between Metarhizium species

**Figure 3:**
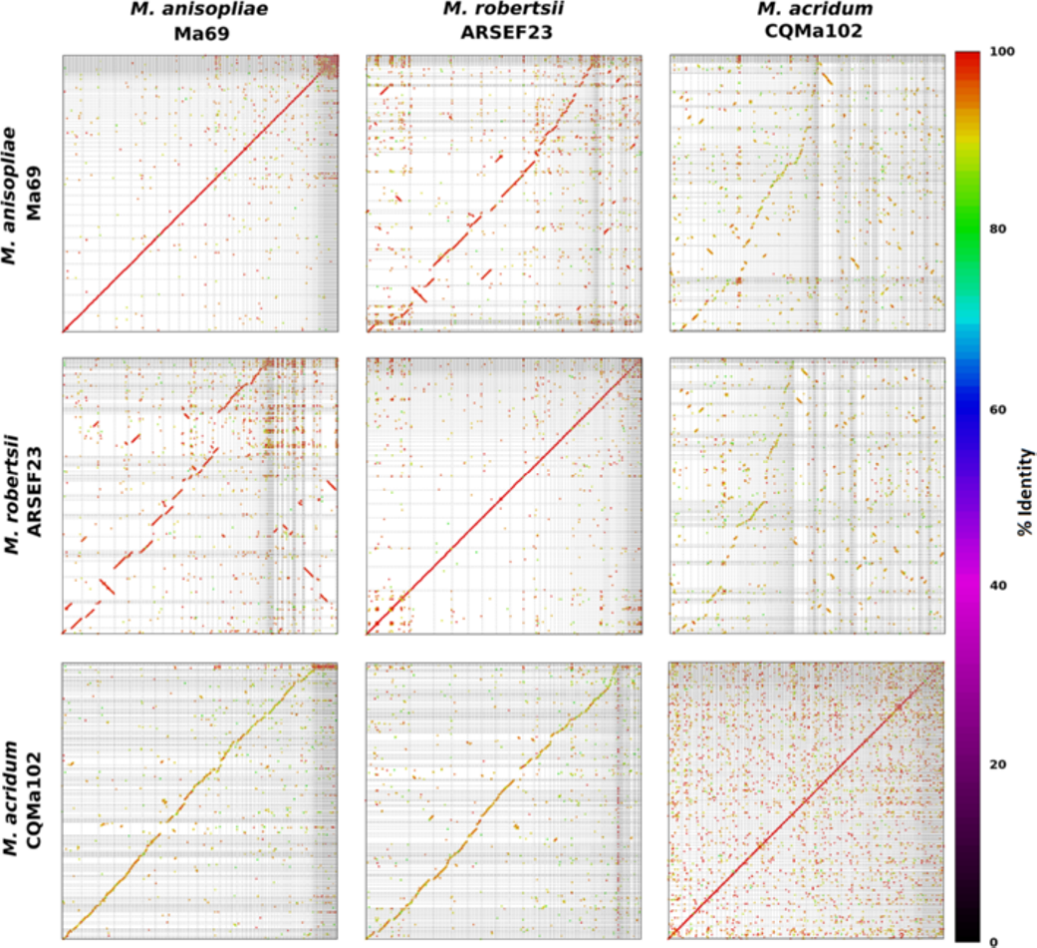
**Whole-genome dot-plot comparison between** ***M. anisopliae*** **(Ma69),** ***M. robertsii*** **(ARSEF23) and** ***M. acridum*** **(CQMa102).** The genome assemblies of all three genomes were compared via MUMMER 3.23 (nucmer). Regions of homology are plotted as diagonal lines or dots which are colour coded for percent identity (colour bar, right). Optimal co-linear order of scaffold sequences was determined by mummerplot (parameter: --fat, MUMMER 3.23) and differs in each pairwise comparison, however match coordinate data for all comparisons is available in Additional file 18.

Synteny dot-plots were generated for pair-wise comparisons between the genomes of Ma69, ARSEF23 and CQMa102. Significant macrosynteny and high levels of sequence homology (≥95% sequence identity) were observed between the genomes of *M. anisopliae* and *M. robertsii* (Figure 3). Macrosynteny was also observed between *M. anisopliae* and *M. acridum* as well as between *M. robertsii* and *M. acridum*, albeit with lower levels of sequence homology (at ~90-95% sequence identity).

### Gene prediction and predicted protein orthology

A total of 11,415 protein-encoding genes were predicted within the *M. anisopliae* Ma69 genome assembly, compared with 10,582 and 9,849 from *M. robertsii* and *M. acridum*, respectively [13]. Orthology relationships between the translated proteins of the three *Metarhizium* species were predicted. While the number of predicted proteins in *M. anisopliae* was greater than those of *M. robertsii* and *M. acridum* (an increase of 833 and 1,566 respectively), most of these appeared to belong to gene families that are expanded in *M. anisopliae* (Additional file 2). A total of 127 proteins from *M. anisopliae* were predicted to have no orthologs in either *M. robertsii* or *M. acridum* (Additional file 3)*.* These proteins were ‘unique-by-orthology’ to *M. anisopliae* and are referred to in this study as ‘divergent’. Additionally, groups of orthologs in which the number of proteins belonging to one species was greater than the other two were classified as ‘expanded’. There were 297 expanded groups containing 603 proteins in *M. anisopliae*, 257 groups containing 562 proteins in *M. robertsii* and 250 groups containing 540 genes in *M. acridum* (Additional file 4).

### Functional annotation of species-specific (divergent) genes, expanded gene families and effector-like proteins in *M. anisopliae,M. robertsii*and *M. acridum*

Orthology relationships between the predicted proteins of the three *Metarhizium* species were used as a basis for predicting ‘divergent’ and ‘expanded’ gene families. Expanded gene families in all 3 species were abundant in proteins with similar Pfam annotations. These were generally related to membrane transport, sugar metabolism, protein kinases, cytochrome p450s, and transcription factors (Additional file 5). Putative functional annotations were assigned to divergent genes by comparison to multiple databases and algorithms, including: BLASTp versus NCBI Protein and Swissprot, gene ontologies (GOs), Interpro, Pfam, SignalP, WolfPsort and BioPerl::SeqStats. Functional annotations were then ‘manually curated’ based on the sum collection of supporting evidence for each gene, with a view, where possible, to intelligibly describe its putative role in pathogenicity. For the purposes of summarizing this analysis, the divergent genes were then sorted into generalized categories based on their putative role and/or function. Of the 127 genes found to be divergent in *M. anisopliae*, 56 (44.1%) contained motifs known to be associated with pathogenicity in other species, 8 (6.3%) had homology to genes in PHIbase [15], 70 (55.1%) were low-molecular weight proteins of ≤30 KDa and 10 (7.87%) had predicted signal peptides (Additional file 6). The divergent genes of *M. anisopliae* were found, compared to its complete gene content, to be relatively more abundant in genes encoding membrane-anchored proteins, transposable elements, or having unknown functions and other functions described in more detail in sub-sections below. Genes encoding DNA/RNA binding factors and degradative enzymes were more abundant in the divergent genes of *M. robertsii* relative to its complete gene content (Additional file 7).

### DNA/RNA binding – transcriptional regulation

Thirteen divergent genes of *M. anisopliae* were broadly characterized as having DNA/RNA binding functions, that is, potentially involved in the regulation of gene expression and intracellular signaling. No signal peptides were identified on these genes. Four of these genes were putative helicases, 2 were putative PIWI-like argonaut/dicer proteins, 4 had endonuclease/exonuclease/phosphatase (EEP) domains, 2 were putative fungal transcriptional factors and one was a putative ribonuclease H homolog.

### Degradative enzymes

*M. anisopliae* had six divergent genes that were grouped in three pairs of paralogs that encoded enzymes with a degradative function. The products of gene paralogs *Ma69_03389*/*Ma69_04038* were putative peptidases (GO: 0008238). Gene paralogs *Ma69_06536*/*Ma69_11051* also encoded putative peptidases, each with a signal peptide and with homology to a *Phytophthora sojae* GIP1-like effector in PHIbase (PHI:653). Gene paralogs *Ma69_00354*/*Ma69_02206* encoded putative subtilisins. *Ma69_00354* in particular was assigned GO terms indicating: serine type endopeptidase (GO: 0004252); active evasion of host immune response via regulation of host complement system (GO: 0042874); alkaline serine protease alp1 (GO: 0005576) and pathogenesis (GO: 0009405).

### Membrane-anchored proteins

Twelve genes, divergent in *M. anisopliae* were classified as encoding membrane-anchored proteins. These genes included a putative arrestin-like G-protein coupled receptor, several membrane-associated proteins and a major facilitator superfamily (MFS) membrane transporter protein. Two genes were homologous to CaNAG4, a membrane transport protein (PHI: 511) and CaMDR1, a multidrug transporter protein (PHI: 26) both found in *Candida albicans*.

### Transposable elements, other genes and unknown genes

Fourteen genes divergent in *M. anisopliae* were identified as transposons and were therefore not considered of interest to this study. Of the remaining divergent genes in *M. anisopliae*, 44 had functions including protein kinases, histidine kinases and heat-shock proteins. Four divergent genes had homology to fungal pathogenicity genes in PHIbase. Two of these were homologous to TOXF from *Cochliobolus carbonum* and two had homology to CTB3 from *Cercospora nicotianae* (PHI: 157 and PHI: 1051 respectively). Due to a lack of homology to the datasets screened, 38 divergent genes, lacked functional annotations and as such their putative biological roles are unknown. Two of these unknown genes had predicted signal peptides.

### Divergent candidate secreted effector proteins in *M. anisopliae*Ma69

Candidate secreted effector proteins are defined here as small protein molecules (≤300 amino acids in length) which are putatively capable of being secreted by the pathogen via the eukaryotic secretory pathway. Secretion to the apoplast relies on the presence of an N-terminal signal peptide and some of these molecules are transferred into plant cells, facilitated by an amino acid motif, downstream of the signal peptide. Of the 127 divergent genes in *M. anisopliae*, 10 were found to encode putative signal peptides. Nine of these were ≤ 300 amino acids in length, four had pathogenicity motifs and these proteins were deemed candidate secreted effector proteins. These candidate secreted effector proteins were assigned to various functional categories including: degradative enzymes (3), membrane anchored proteins (2), other (2) and unknown genes (2).

### Repetitive DNA content and repeat-induced point mutation (RIP)

**Figure 4:**
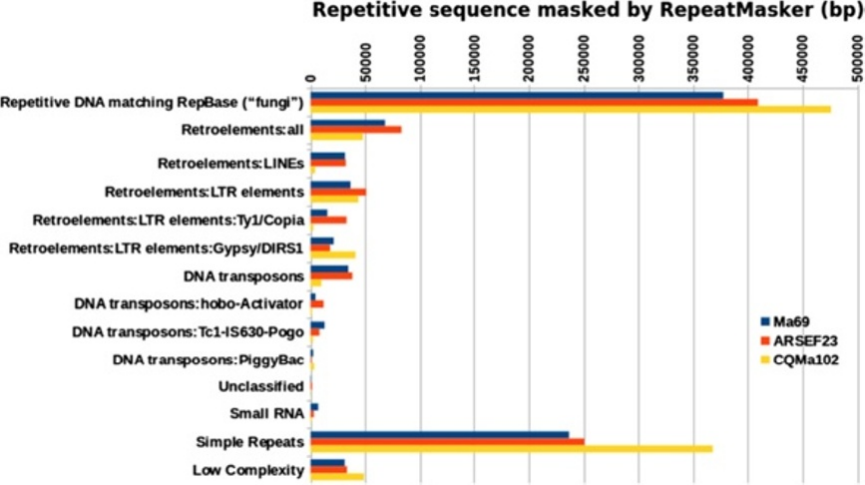
**Comparison of the amount of sequence represented by various repeat classes across the 3** ***Metarhizium*** **species,** ***M. anisopliae*** **Ma69,** ***M. robertsii*** **ARSEF23 and** ***M. acridum*** **CQMa102, as determined by RepeatMasker comparison to RepBase.**

Repetitive DNA analysis was performed on the scaffold sequences of *M. anisopliae* and the published reference genomes of *M. robertsii* and *M. acridum*. Repetitive sequences were predicted *de novo* and RIPCAL was used to determine genome-wide dinucleotide frequencies and to quantify RIP-like polymorphisms (SNP mutation biased towards CpA → TpA or its reverse complement TpG → TpA) within alignments of each repeat family (using the deRIP consensus as the model sequence for comparison) [16, 17]. To facilitate comparison of repeat types between *Metarhizium* spp., their respective genomic matches to characterized repeat sequences in RepBase [18] are also shown in Figure 4. Overall, the repetitive contents of the genome of *M. anisopliae* Ma69 and *M. robertsii* ARSEF23 were similar and relatively low in comparison to other Pezizomycotina. In contrast, the *M. acridum* genome assembly contained approximately twice as much repetitive DNA. In total, 2.4% of the genome assembly was predicted to be repetitive for *M. anisopliae*, 2.12% for *M. robertsii*, and 4.42% in *M. acridum*. In the three *Metarhizium* genomes, the most abundant repeat type was simple/low-complexity repeats, followed by retroelements and DNA transposons. The increased repetitive content of *M. acridum* was largely due to increased amounts of simple/low-complexity sequences. Interestingly, *M. acridum* was also relatively depleted in transposons and though maintaining a comparable level of LTR retroelements was relatively deficient in LINEs and DNA transposons.

Repeat families were scanned for repeat-induced point mutation (RIP)-like dinucleotide changes using two methods. The first involved genome-wide comparison of dinucleotide frequencies between *de novo*-identified repetitive sequences and non-repetitive sequences (Additional file 8). The RIP index TpA/ApT, which measures the frequency of the common RIP-product TpA versus a non-RIP like control ApT, was 1.02 within repeats of *M. anisopliae* and *M. robertsii* and 1.31 in *M. acridum*. Dinucleotide frequency analysis also showed an overall increase in TpA dinucleotides in *M. acridum* relative to *M. anisopliae* and *M. robertsii* and a corresponding depletion of the RIP-target dinucleotides CpA and TpG (Additional file 8).

The second RIP-quantitation method used the RIPCAL alignment-based method [16] versus a ‘deRIPped’ consensus of each family as a reference for comparison [17]. RIP mutation statistics for all repeat families in all three species are tabled in Additional file 9. RIP levels were on the whole relatively low in all three species, however all three species had a *rid* (cytosine-5 methyltransferase) homolog [19]. RIPCAL analysis showed that *M. anisopliae* and *M. robertsii* had similar total levels of RIP mutations and both species exhibited elevated levels of mutation of CpA dinucleotides (as well as CpT in some cases) typical of RIP in the Pezizomycotina in some repeat families. It should be noted that RIP mutation requires a minimum repeat length and we would not expect it to occur in repeats less than 400 bp in length [20, 21]. However assemblies containing short scaffolds such as those used in this study means these *Metarhizium* assemblies are likely to contain a high number of short repeat families which are incomplete versions of full length repeats. A total of 40 repeat families of *M. anisopliae*, ranging in average repeat family length of 47 to 375 bp, had CpA↔TpA (RIP-like) dominance scores of 1 or greater (Additional file 9 - summary). In *M. robertsii*, 12 repeat families had a similar RIP-like dominance score, with lengths ranging from 54 to 173 bp. In contrast, *M. acridum* had 56 repeat families with a RIP-like dominance score ≥1, lengths ranging from 45 to 1225 bp. Interestingly, the total number of RIP-like mutations in *M. robertsii* (7624) (relative to ‘deRIPped’ repeat consensus sequences), was approximately five times fewer than those of *M. anisopliae* and *M. acridum* (34960 and 39934 respectively).

### Mating (MAT) type gene analysis

**Figure 5:**
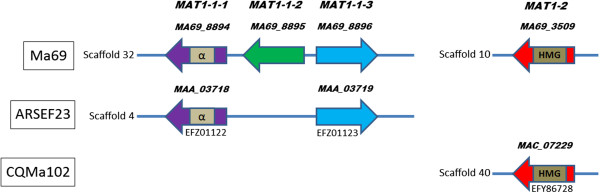
**Presence and absence of mating type genes in** ***M. anisopliae*** **Ma69,** ***M. robertsii*** **ARSEF23 and** ***M. acridum*** **CQMa102.**

Analysis of orthologous relationships between the genes of the three *Metarhizium* species in this study identified incongruence in the number and presence of MAT genes (Figure 5). Ma69 had three genes putatively identified as *MAT 1-1-1* (*MA69_8894*), *MAT1-1-2* (*MA69_8895*) and *MAT1-1-3* (*MA69_8896*) with no orthologous pairs in *M. acridum*, and orthologs of *MAT 1-1-1*(*MAA_03718*) and *MAT1-1-3* (*MAA_03719*) in *M. robertsii*. Ma69 also had one gene (*MA69_3509*) putatively identified as *MAT1-2* which had an ortholog (*MAC_*07229) in *M. acridum*, but not in *M. robertsii*.

**Figure 6:**
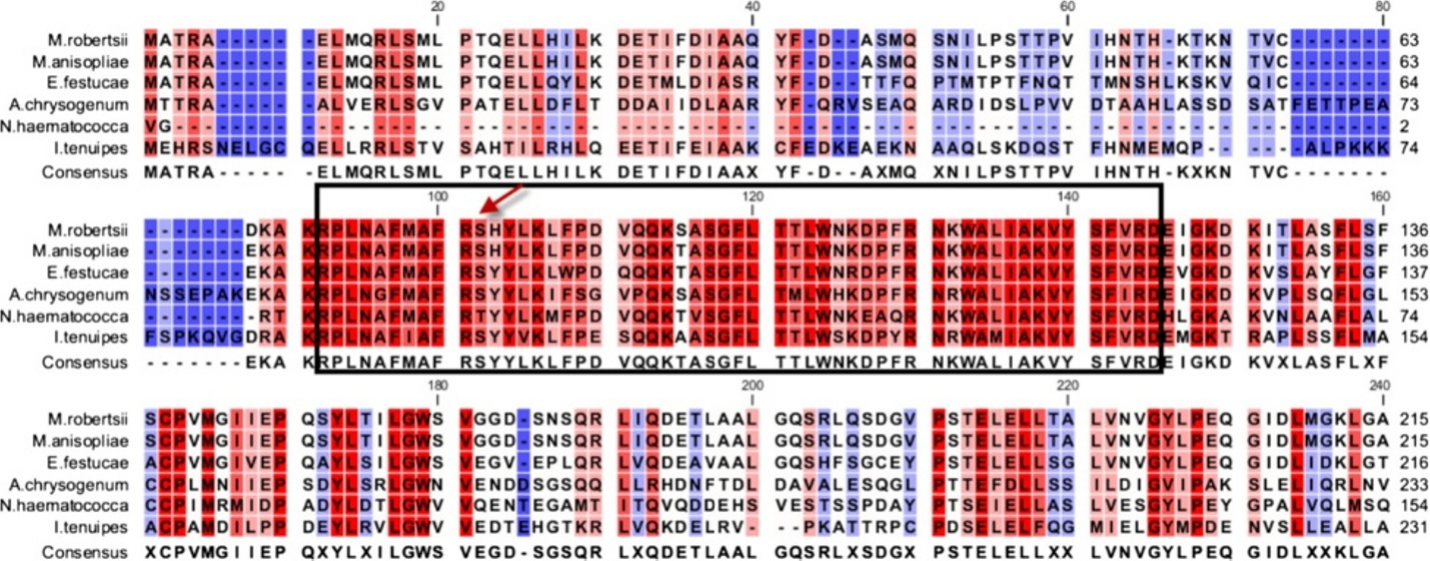
**The MAT1-1-1 sequence of** ***M. anisopliae*** **(Ma69) was analysed by BLASTp and the sequences of** ***M. robertsii*** **(EFZ01122),** ***Epichloe festucae*** **(ACN59937),** ***Acremonium chrysogenum*** **(CAQ42990),** ***Nectria haematococca*** **(XP_003052790) and** ***Isaria tenuipes*** **(BAC67541) were downloaded and aligned (partial sequence shown here).** The conserved alpha box is boxed and highlighted in red and an arrow designates the conserved intron.

**Figure 7:**
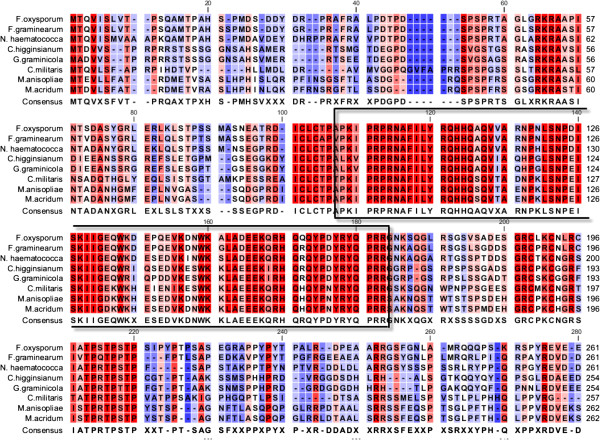
**The MAT1-2 sequence of** ***M. anisopliae*** **(Ma69) was analysed by BLASTp and the sequence hits** ***Fusarium oxysporum*** **(EGU84400),** ***F. graminearum*** **(XP_385327),** ***Nectria haematococca*** **(XP_003051581),** ***Colletotrichum higginsianum*** **(CCF36618),** ***Glomerella graminearum*** **(EFQ33384),** ***Cordyceps militaris*** **(EGX93214) and** ***M. acridum*** **(EFY86728) were downloaded and assembled (partial sequence shown).** The conserved HMG domain is boxed and highlighted in red.

The four putative *MAT* genes from Ma69 were subjected to BLASTp analysis and were found to have near identical sequence homology to ascomycetous mating type (*MAT*) genes (Additional file 10). The top ten sequence hits for each MAT gene were aligned to confirm the identity of the genes and identify conserved domains (Additional file 11). The *M. anisopliae MAT1-1* alpha box domain [Pfam: PF04769] was confirmed by alignment to known *MAT1-1* sequences and carries a conserved intron (Figure 6) [22, 23]. The *M. anisopliae MAT1-2* HMG-box domain [Pfam: PF00505] was also confirmed by alignment with other *MAT1-2* sequences and also carries a conserved intron (Figure 7) [22, 24]. The presence of both *MAT* idiomorphs in the genome of the *M. anisopliae* isolate sequenced indicates it to be putatively homothallic.

### Prediction of secreted effector protein candidates via species-specificity and secretion predictions

To our knowledge, pathogenicity effector-like motifs have not yet been examined in *Metarhizium* species. Some experimentally-validated pathogenicity effector proteins exhibit short conserved amino-acid motifs (Additional file 12), that in some cases have been demonstrated to facilitate host-cell import [25]. We combined protein matches to these known ‘effector motifs’, with predictions of signal-peptides (which infer extracellular secretion of the protein), to arrive at a conservative set of predicted secreted effector protein candidates.

In total, 1,620 proteins in *M. anisopliae* Ma69 contained sequences matching to a motifs associated with pathogenicity in other fungal species (Additional file 13). The most abundant motifs found were [YWF]XC, [LI]XAR and RXLR. These motifs are short (generally 3–4 amino acids), thus a substantial number of false-positive matches to proteins would be expected, in particular in long, intracellular proteins unrelated to pathogenicity. However these motifs were only considered and found in high numbers among groups of genes that had been previously bioinformatically-filtered for protein properties relevant to pathogenicity. Potential pathogenicity genes were identified using orthology as a basis for predicting species-specific (‘divergent’) genes in all 3 species, which are strong candidates as determinants of their respective differences in host-range phenotype. Seven amino acid motifs known to be associated with pathogenicity proteins were found to match several divergent genes of *M. anisopliae, M. robertsii* and *M. acridum* (Additional file 14).

Furthermore, 1,295 proteins were predicted to be secreted in *M. anisopliae* Ma69 (Additional file 15). We identified 242 of these proteins that we term ‘candidate effectors’, which both contained a pathogenicity effector motif and were predicted to be secreted (Additional file 16). Among these 242 candidate effectors, 6 motifs were found, in descending order of abundance: [YFW]XC (166 proteins), [LI]XAR (54), RXLR (18), CHXC (2), KECXD (1) and YXSL[RK] (1).

## Discussion

*M. anisopliae* is a fungus with significant commercial and industrial applications as a biopesticide. The host and environmental range of *M. anisopliae* is regarded as entomologically cosmopolitan, compared to that of *M. acridum* which is host-specific. Recent molecular analysis of the *M. anisopliae* complex has proposed its division into nine taxa, or distinct species, which individually may have narrower host specificities [5, 8]. In a multigene phylogeny, *M. anisopliae* clustered with three other species *M. pingshaense*, *M. robertsii* and *M. brunneum* forming a distinct clade (PARB) [5]. Whether or not these represent discrete biological species or merely *formae specialis* is not known, as *M. anisopliae* has no known teleomorph [6] and the basis of its assumed asexuality has not been determined. Furthermore, advancing the currently poor understanding of the underlying mechanisms of genetic exchange could have important implications for understanding the persistence of traits of biopesticides in commercial applications.

Given that the isolate ARSEF23 sequenced as *M. anisopliae* has subsequently been re-classified as *M. robertsii*, and is sufficiently divergent from *M. anisopliae* [5, 9], an alternative genome reference is required for this important biocontrol fungus to differentiate it from and complement the existing *Metarhizium* genomic resources. Thus, we sequenced the genome of *M. anisopliae* isolate Ma69 using Illumina short-read sequence data, producing an annotated, *de novo* draft genome assembly of 577 scaffolds with an N50 of 11 and an N50 length of 1.24 Mb. The whole genome assembly and its predicted gene content was compared to the published genomes of *M. robertsii* (ARSEF23) and *M. acridum* (CQMa102) (Table 1), with particular focus on the species-specific genes of *M. anisopliae* Ma69, mating type and bioinformatic prediction of its candidate secreted effector proteins.

### Whole genome synteny, homology and divergent genes in *M. anisopliae*

The comparative genomics of *M. robertsii* and *M. acridum* has been comprehensively examined [13] and we have refrained from duplicating previous efforts, except to include and compare *M. anisopliae* in that context. The previously published genome analysis of *M. robertsii* [13] showed syntenic conservation with gene clusters of *M. acridum* but did not present any whole-genome scale synteny comparisons. This was likely due to the poorer contiguity (as indicated by N50, Table 1) of the *M. acridum* genome assembly, which would prevent whole-genome scale synteny from being accurately observed. In this study, whole genome comparisons including the new assembly of *M. anisopliae,* in which the largest scaffolds are of a comparable level of contiguity with those of *M. robertsii*, indicate a generally macrosyntenic conservation pattern (Figure 3, Additional file 17 and Additional file 18). A low level of intra-chromosomal rearrangement is also observed, however this is similar to levels of degraded macrosynteny between other Pezizomycotina species of the same genus, such as previously observed between the Aspergilli [26]. We found that both *M. anisopliae* and *M. robertsii* had nucleotide sequence identities ≥ 90% to *M. acridum*, but sequence identity to each other was ≥ 95%, confirming that *M. anisopliae* and *M. robertsii* are more closely related to each other than to *M. acridum*.

A total of 11,415 proteins were predicted in the *M. anisopliae* genome assembly*,* 833 and 1,566 more than *M. robertsii* and *M. acridum* respectively. We examined the orthology of genes within the three *Metarhizium* species and found 127 genes in Ma69 that had no orthologs in *M. robertsii* and *M. acridum*, which we refer to in this study as ‘divergent’ genes. We also defined a set of ‘expanded’ genes in which the number of inparalogs of a single species was greater than corresponding outparalogs in the other two species, of which for *M. anisopliae* there were 603 expanded genes within 297 ortholog groups.

### Repetitive DNA, TE classes and RIP analysis

Repetitive sequences are associated with transposable elements (TEs) which play a central role in the evolutionary restructuring of fungal genomes due to their ability to move within the host genome, causing a range of mutations [27]. Excluding deleterious insertions, the mutational activity of TEs may promote genetic diversity and speed up adaptative evolution in the host [28]. Class I (retroelements) use a ‘copy and paste’ mechanism to transpose via the reverse-transcription of an RNA intermediate and include long terminal repeats (LTRs), non LTRs, and long and short interspersed nuclear elements (LINEs and SINEs, respectively [27, 29]. Class II TEs (DNA transposons) ‘cut and paste’ directly through a DNA form, using the enzyme transposase [27, 29]. Earlier, we detailed our observations of phenotypic variation within single spored cultures of *M. anisopliae* (Figure 2)*.* Due to their ability to directly excise from DNA and re-insert elsewhere in the genome, DNA transposons can generate a wide range of DNA sequence variation which may result in phenotypic changes and may account for the variety of cultural phenotypes observed. Alternatively, the abundance of Class II TEs in *M. anisopliae* may also affect cultural morphology, as evidence by LINE mediated mutations causing changes to conidial pattern formation in *Magnaporthe grisea* [30].

At the gene level, insertion of TEs either in or adjacent to genes may cause partial or total gene inactivation, resulting in new phenotypes [27, 29]. On a genome-wide scale, TEs may be associated with large scale chromosomal modifications such as deletions, inversions and translocations [27]. To prevent or minimize potential deleterious effects of TEs, some fungi possess a gene silencing mechanism known as repeat induced point (RIP) mutation which targets duplicated DNA sequences > 400 bp long with > 80% shared identity [31]. Comparisons of the repeat content of the three *Metarhizium* spp., using reciprocal blast clustering, revealed surprisingly few common repetitive sequences. Further analysis of RIP-like polymorphism in all three *Metarhizium* spp. confirmed a common dinucleotide bias for CpA which conforms with expectations for species of the Pezizomycotina. As found in a number of species of Pezizomycotina [32], all three *Metarhizium* species examined here possessed a rid homolog which is essential for RIP and although the function of these genes is untested, we assume that RIP is active in all three species. In silico analysis of repetitive DNA and RIP across the three *Metarhizium* spp. indicated that repetitive content in *M. anisopliae* and *M. robertsii* was low at around 2% of genomic DNA and doubled in *M. acridum* at around 4%. Dinucleotide frequency analysis supports active RIP in all 3 species, with elevated frequencies of the RIP-product TpA and depleted levels of the primary RIP targets CpA and TpG within their respective repetitive DNA complements. *M. acridum* also exhibited a significant increase in TpA and decrease in CpA and TpG relative to the other two species, perhaps indicative of its higher transposon content. However a more complex picture emerges after alignment-based RIPCAL analysis, which supports similar levels of RIP mutations between *M. anisopliae* and *M. acridum*, with a predicted 5-fold decrease in RIP-like polymorphism in *M. robertsii*. We speculate that while *M. anisopliae* and *M. robertsii* appear to contain similar levels of repetitive DNA, RIP activity may be greater in *M. anisopliae* than in *M. robertsii*. Higher numbers of RIP-like polymorphism in *M. anisopliae* and *M. acridum* may suggest a slightly greater adaptive potential compared to *M. robertsii*, as pathogenicity-related genes have previously been demonstrated to be affected by RIP mutations leaking outwards from flanking repetitive DNA [33, 34].

### MAT gene orthology

Sexual function in filamentous ascomycetes is determined by mating type loci (*MAT*) which has been extensively described [35–37]. Typically, the single locus determining mating behaviour between mating partners contains different genes which are not allelic and are therefore known as idiomorphs [35, 36]. In filamentous ascomycetes (Pezizomycotina), *mat* genes encode DNA binding motifs (high-mobility group (HMG) boxes and α domains) [24] and are responsible for the control of both mating and incompatibility, cell-cell recognition and recognition between nuclei [35]. The lack of an observed sexual lifestyle in *Metarhizium* species may be the result of a loss of gene function, the lack of an opposite mating type, or merely the inability to induce a teleomorph under laboratory conditions. Functional *MAT* genes have been discovered in other ascomycetes previously assumed to be asexual (formerly known as Deuteromycetes) but the potential for a sexual cycle in these remains enigmatic [37–40] and indeed, its manifestation may occur on a continuum of sexuality in fungi, ranging from common to rare [41].

We identified both *MAT1-1* and *MAT1-2* idiomorphs in *M. anisopliae*, indicating that it is putatively homothallic and possibly capable of sexual reproduction. The Ma69 *MAT1-1* idiomorph encodes three proteins: *MAT1-1-1*, an α domain protein; *MAT1-1-2*, an amphipathic α-helical protein; and *MAT1-1-3*, an HMG box protein [42]. At this point, the functionality of these genes in *M. anisopliae* is not known, however sequence analysis has yielded a high level of conservation of alpha box and HMG domains with other ascomycetes. This warrants further investigation, including gene expression and/or transformation of closely related teleomorph species.

The *M. acridum* isolate CQMa102 possessed the *MAT1-2* gene but lacked the *MAT1-1* idiomorph. The *MAT1-2* gene encoded an HMG-domain protein that was highly conserved with *M. anisopliae* and other ascomycetes. Until an opposite mating type can be found and functionality is confirmed, heterothallism in *M. acridum* is deemed putative at this juncture. In contrast to both *M. anisopliae* and *M. acridum*, the *M. robertsii* isolate ARSEF23 possessed an incomplete *MAT1-1* idiomorph and lacked an ortholog to *MAT1-2*. Absence of the *MAT1-2* idiomorph would indicate that the isolate sequenced was *MAT1-1* heterothallic, however again, in the absence of an opposite mating type and without confirmation of functionality, heterothallism is deemed putative. The relatively similar extents of RIP-like polymorphism in *M. anisopliae* and *M. acridum*, given the putative homothallism of *M. anisopliae*, is perplexing. RIP occurs only during meiosis, and as such, evidence of RIP may indicate the occurrence of meiosis in an ancestral or cryptic sexual stage, or alternatively, the existence of a similar process in vegetative cells [27]. The results of RIP analysis for *M. robertsii*, which indicate approximately 5 times less RIP than in the other *Metarhizium* spp., lends support to impaired or less frequent sexual activity in this species.

The missing MAT1-1-2 gene in *M. robertsii*, a phenomenon also observed in *Pyrenopeziza brassicae* and *Cochliobolus heterostrophus*, two heterothallic species with sexual stages [37] is intriguing. A functional MAT1-1-1 gene is critical to mating identity, sexual development [42] and vegetative incompatibility [43] as the alpha box domain, which is subsequently processed into mature pheromone molecules is located here [44]. However the requirement for a functional MAT1-1-2 is less clear. In the aforementioned example of *P. brassicae*, the absence of MAT1-1-2 does not impede out crossing, indeed the teleomorph is found naturally in oilseed rape [45]. In another example, *Neurospora crassa*, the homologs of MAT1-1-2 and MAT1-1-3 (reported as matA-2 and matA-3, respectively) were reported to be non-essential for mating or ascospore production [43, 46], however their expression increased the efficiency of sexual development [43]. It was also reported that homologs of MAT1-1-2 and MAT1-1-3 in *Podospora anserina* (reported as α-helical genes and HMG-1 genes, respectively) were not required for mating but were required for sexual development and biparental progeny, as mutations in the α-helical gene lead to barren fruiting bodies [37, 47]. The evidence suggests that while MAT1-1-1 is crucial to mating, the MAT1-1-2 gene may have a supporting role in some ascomycetes in the successful development of sexual bodies, post mating, while in other species, its absence does not impede the development of fit progeny. The effect of the absence of MAT1-1-2 in *M. robertsii* is unknown at this point, however it is intriguing that its closely related sibling species, *M. anisopliae* possesses the full complement of MAT1-1 genes. In future, the discovery of isolates of *M. robertsii* and *M. acridum* with complementary MAT idiomorphs followed by evolutionary analysis of these idiomorphs may help to further our understanding of the role of MAT1-1-2 in the *Metarhizium* species complex, and the potential effect on genetic exchange and perhaps, the observable phenotypic variation between cultures.

### Candidate secreted effector proteins

In this analysis, we identified a suite of genes which have the characteristics of effector proteins. Effector proteins are defined as molecules produced by a pathogen, which can alter host cell structure or function, thereby facilitating infection and/or initiating defense mechanisms [48]. Putative effector proteins are characterized in this study as being ≤ 300 amino acids in length and have a predicted signal peptide, which would facilitate secretion into the pathogen’s extracellular space. In addition to these characteristics, effector predictions may also be supported by matches to proteins with previously identified pathogenicity motifs.

Proportionately, the genome of *M. anisopliae* had similar numbers of putative secreted effector-like proteins to *M. robertsii*, and more secreted effector-like proteins than *M. acridum* suggesting a similar capability or role of secreted proteins between *M. anisopliae* and *M. robertsii*. Ten predicted secreted effectors in *M. anisopliae* were divergent from *M. robertsii* and *M. acridum*. In particular, 3 of these divergent secreted effectors were determined to be putative degradative enzymes, which is of significant biological interest to *Metarhizium* biopesticide research and warrants further investigation to identify their function and potential effect on host range.

We found six motifs known to be associated with pathogenicity in other species, to be present in 242 candidate secreted effector-like proteins of *M. anisopliae.* Of these, 166 matched [YFW]xC, 54 matched [LI]XAR and 18 matched RXLR and represent the first analysis of effector-assosciated motifs in an entomopathogenic fungus. The best characterized of these motifs is the RXLR motif found in the oomycete *Phytophthora infestans* [49], although conserved RXLR motif effectors have yet to be observed in fungi. In pathogenicity effectors of *P. infestans*, the RXLR motif is found adjacent to the signal peptide, on the N-terminal end of the mature cleaved protein, and has been shown to facilitate translocation of the effector protein across the host membrane into the host cell. The [LI]XAR motif was identified in effector proteins secreted by the rice blast pathogen *Magnaporthe oryzae,* however the function of this motif is unknown [50]. As such, the function of these motifs in *Metarhizium* remains speculative.

While the high number of [YFW]XC motifs among the two predicted sets of genes, divergent and effector candidates, may simply be an artifact of random background matches to this short, three residue motif, there is some evidence suggesting that there may be an important, albeit, still unknown role for [YFW]XC motifs in secreted proteins. The [YFW]XC motif was first reported in the powdery mildew fungus *Blumeria graminis* [51]. It was predominantly expressed in the haustoria and was also over-represented among its predicted secretome [51]. Since then, the [YFW]XC motif has also been discovered among effector candidates from the haustoria-producing rust fungi *Puccinia graminis* f.sp. *tritici, P. striiformis* f.sp. *tritici* and *Melampsora larici-populina* (as cited by Pedersen). *M. anisopliae* also produces haustoria *in vitro* under nutrient deprivation [52] however the link between the [YFW]XC motif and production of haustoria is still yet to be resolved. The function of the [YFW]XC motif still remains unclear, however it may have arisen from an extracellular RNase ancestor [53] and may be involved in establishing disulphide bridges with a C-terminal cysteine residue (Thordal-Christensen pers. comm.) thereby assisting protein folding and enhancing extracellular stability. Pedersen et al., [53] hypothesize that a secreted fungal ribonuclease appears to be the common origin of many of their candidates for secreted effector proteins (CSEPs). They suggested that some of these CSEPs could still be involved in interactions with host RNAs and modulate host immunity via this route. They also go on to suggest that extracellular ribonucleases are very stable molecules, resistant to proteolytic degradation, thereby providing a rigid scaffold ideal for evolving an effector arsenal, in which exposed loop regions subjected to positive selection allow diversification and evasion of host recognition. The structural conservation among effector candidates from diverse plant pathogenic species supports the hypothesis of an ancient common ancestor. The entomopathogenic *M. anisopliae* is likely to have arisen from a plant-pathogenic predecessor [13] and the discovery of these motifs in *Metarhizium* is consistent with the high level of conservation and putative functional requirement of these for pathogenicity.

## Conclusions

### Genomics, phenotypic variability and biopesticides

The key to successful commercialization of biopesticides is the identification and selection of virulent microbial isolates which are amenable to industrial scale production and formulation [54]. Information from comparative genomics studies can be used to identify genes which may contribute to isolate virulence and fitness as well as other characteristics which affect cultural variability and stability of potential biopesticides.

The discovery of both MAT idiomorphs in *M. anisopliae* raises more questions about its perceived asexuality, the potential pathways of genetic exchange in this species, impact on virulence, and implications for industrial applications. Understanding the molecular basis of mating genes not only gives insight into fundamental processes such as evolution of homothallism, heterothallism and asexuality, but also facilitates research on ascomycetous species of industrial interest and subsequent applied aspects [55]. Proof of function or not in these genes, will enhance the understanding of genetic exchange pathways in this species.

Overall, the repetitive contents of the genome of *M. anisopliae* Ma69 and *M. robertsii* ARSEF23 were similar and relatively low in comparison to other Pezizomycotina. In contrast, the *M. acridum* genome assembly contained approximately twice as much repetitive DNA. RIP levels were on the whole relatively low in all three species, however all three species had a *rid* (cytosine-5 methyltransferase) homolog and we observed SNP polymorphism consistent with active RIP in all three species. *M. anisopliae* and *M. robertsii* had similar total levels of RIP –like mutations and both species exhibited elevated levels of mutation of CpA dinucleotides (as well as CpT in some cases) typical of RIP in the Pezizomycotina in some repeat families. Interestingly, the total number of RIP-like mutations in *M. robertsii* was approximately five times fewer than those of *M. anisopliae* and *M. acridum*, lending support to impaired or less frequent sexual activity in this species. RIP occurs only during meiosis, and as such, evidence of RIP in these species may indicate the occurrence of meiosis in an ancestral or cryptic sexual stage, or alternatively, the existence of a similar process in vegetative cells.

The availability of an annotated whole genome sequence for *M. anisopliae* adds value to the already published genomes of *M. robertsii* and *M. acridum*; however in and of itself, represents a significant resource for future research into this agriculturally important fungal biopesticide. The nomenclature of the species *M. robertsii* needs to be widely adopted immediately to prevent further confusion with *M. anisopliae*, therefore the publication of the genome reference of *M. anisopliae* will serve as a valuable reference to differentiate it from *M. robertsii.*

## Methods

### Origin and culture of fungal strain Ma69

An Australian isolate of *M. anisopliae* known to be pathogenic to aphids was selected for whole genome sequencing. Originally, *M. anisopliae* isolate BRIP 53293 was isolated from soil in the Kingaroy region, Queensland and was obtained from the Queensland Department of Employment, Economic Development and Innovation (DEEDI). A single spore culture of BRIP 53293 (BRIP 53293 EFD 69 SSC31) was prepared on Sabouraud Dextrose Agar (SDA) before being transferred to Sabouraud Dextrose Broth (SDB), where cultures were shaken (150 rpm) at 25°C for 5 days prior to DNA extraction. For brevity, isolate BRIP53293 EFD69 SSC31 will be referred to hereafter as Ma69.

### Genome sequencing and assembly

Genomic DNA was extracted from the single-spored fungal culture using NucleoSpin Plant II (Machery Nagel) DNA extraction kit as per the manufacturer’s instruction. The ITS region of ribosomal DNA (rDNA) was sequenced [56] and subjected to BLAST [57] analysis to confirm the identity of the isolates. Sufficient genomic DNA was then prepared for paired-end and mate-pair sequencing at the Australian Genome Research Facility (AGRF), Brisbane according to Illumina protocols. Raw read sequences were trimmed via cutadapt [58] for: Illumina adaptor sequences; PCR primer and barcode contaminant sequences; homopolymers and runs of unknown bases > 5 bp; base quality > Q30 and; a minimum read length after trimming ≥ 50 bp. Reads with a discarded pair (i.e. with a length of < 50 bp after trimming) were removed from paired-end datasets, but were retained as singleton reads.

### De novo assembly of Ma69

Paired-end reads were assembled *de novo* with Velvet (version 1.1.2) [59]. The k-mer length parameter was tested between 20 and 70 bp in 2 bp increments and optimized for minimal N50 and maximal N50 length. Velvet paired-end scaffolds were then scaffolded with reads from the 3 kb mate-paired library via SSPACE (version 1.1) [60].

### Comparative genomics: whole assembly alignment

Whole genome alignments between the genome assemblies of Ma69, ARSEF23 and CQMa102 were performed with MUMMER 3.23 [61], using the nucmer algorithm with the –mum parameter. Dot-plots were generated with mummerplot with the –filter, -colour and –fat parameters.

### Gene prediction

Putative protein-coding regions of the Ma69 assembly were predicted with GeneMark-ES [62]. Predicted gene models were filtered for a minimum protein product length of 50 amino acids. Predicted genes encoding products < 50 amino acids in length were discarded.

### Protein family classification and orthology

Predicted proteins of Ma69 were compared for orthology to the ARSEF23 and CQMa102 published protein datasets, obtained from the NCBI NR protein database. Orthologous relationships between genes of these three species were inferred with ProteinOrtho4 using the –pair and –selfblast parameters [63]. A list of divergent genes specific to each species was generated according to non-orthology. In order to assign putative functions to the species-specific gene datasets, the datasets were screened and compared to a number of databases and were then manually-curated based on the sum collection of evidence for each gene. The genes were then sorted into generalized categories based on their putative role/function. The whole proteome datasets for each species were screened via EMBOSS for sixteen motifs known to be involved in fungal pathogenicity (Additional file 9). Homology to PHIbase (v 3.2) protein sequences were assessed by BLASTp (e-value threshold 1e-5). The best BLAST hit was assigned on the basis of highest bit score and the disruption phenotype was recorded. Gene Ontology (GO) terms were assigned to the species-specific gene translated sequences via Blast2GO (default settings with BLASTp, Annex augmentation). Interproscan was also enabled within Blast2GO assigning additional GO term, functional domain and structure annotations. Secretion, sub-cellular localization, molecular weight and iso-electric point were calculated for each species-specific dataset via SignalP 3.0 [64], WolfPsort [65] and EMBOSS IEP [66]. Amino acid compositions were calculated using the SeqStats BioPerl module [67].

### Repetitive DNA analysis

*De novo* prediction of repetitive sequences of Ma69 and the published reference genomes of ARSEF23 (*M. robertsii*) and CQMa102 (*M. acridum*) were generated using RepeatScout [68] while overlapping and redundant consensus repeats were combined via CAP3 [69]. These *de novo* repeats were mapped to the genome assembly via RepeatMasker [70]. For the purpose of comparison of characterised repeat types, RepeatMasker was also run versus each *Metarhizium* spp. assembly using RepBase sequences corresponding to the “fungi” taxon. Multiple alignments of repeat families were generated by RIPCAL [16] using ClustalW [71, 72]. deRIP [17] was used to predict the pre RIP consensus of each aligned repeat family. RIPCAL [16] was run on each repeat family alignment using the deRIP consensus as a model sequence for comparison. Repeats for all species were analyzed using TEClass [73] to predict the most probable class for each repeat family. Repeats common between all species were identified using proteinortho4 using the ‘--p blastn’ parameter [63].

### Mating (MAT) type gene analysis

MAT genes were identified by homology to characterized mating-type sequences using BLASTP [57]. The amino-acid sequences top ten BLASTp hits for each putative Ma69 MAT protein were obtained from NCBI and multiple-alignment on these was performed in CLC bio Genomics Workbench v4.0 (CLC bio, Denmark).

### Search for known effector-associated motifs

Proteins were assessed for the presence of domains known to be associated with effectors outlined in Additional file 9 using EMBOSSpreg v 6.5.7 (http://emboss.sourceforge.net/apps/release/6.3/emboss/apps/preg.html).

## Electronic supplementary material

Additional file 1: **Top ten BLAST hits for Ma69 (complete sequence ITS1; 5.8S rRNA gene; ITS2).** (PDF 11 KB)

Additional file 2: ***Metarhizium*** **ortholog table: Orthology relationships between the translated proteins of the three** ***Metarhizium*** **species were predicted with ProteinOrtho4 using the –pair and –selfblast parameters.** (XLSX 368 KB)

Additional file 3: ***Metarhizium*** **species-specific gene summary: A total of 127 proteins from** ***M. anisopliae*** **were predicted to have no orthologs in either** ***M. robertsii*** **or** ***M. acridum.*** These proteins were ‘unique-by-orthology’ to *M. anisopliae* and are referred to in this study as ‘divergent’. (XLS 90 KB)

Additional file 4: **Orthologs and expanded genes: Groups of orthologs in which the number of proteins belonging to one species was greater than the other two were classified as ‘expanded’.** There were 297 expanded groups containing 603 proteins in *M. anisopliae*, 257 groups containing 562 proteins in *M. robertsii* and 250 groups containing 540 genes in *M. acridum.* (XLS 3 MB)

Additional file 5: **Pfam functional annotations assigned to** ***Metarhizium*** **proteins.** (XLS 6 MB)

Additional file 6: ***Metarhizium*** **species-specific gene summary: Putative function annotations were assigned to divergent genes by comparison to multiple databases and algorithms, including: BLASTp versus NCBI Protein and Swissprot, gene ontologies (GOs), Interpro, Pfam, SignalP, WolfPsort and BioPerl::SeqStats.** Functional annotations were then ‘manually curated’ based on the sum collection of supporting evidence for each gene, with a view to intelligibly describe its putative role in pathogenicity. For the purposes of summarizing this analysis, the divergent genes were then sorted into generalized categories based on their putative role and/or function. (XLS 90 KB)

Additional file 7: **Functional classification of divergent gene in** ***M. anisopliae, M. robertsii*** **and** ***M. acridum*** **: Gene annotations listed for which amino acid translations (predicted proteins) were found by reciprocal-best hit analysis via Proteinortho between the three species.** (PDF 51 KB)

Additional file 8: **Summary of RIP dinucleotide analysisof** ***de novo*** **-identified repetitive seqeunces and non-repetitive sequences.** (XLS 17 KB)

Additional file 9: **RIP mutation statistics for all repeat families in all three species: Repeat families were scanned for repeat-induced point mutation (RIP)-like dinucleotide changes using two methods.** The second RIP-quantitation method used the RIPCAL alignment-based method versus a ‘deRIPped’ consensus of each family as a reference for comparison. (XLSX 369 KB)

Additional file 10: **BLASTp identification of putative MAT genes from** ***M. anisopliae*** **isolate Ma69.** (PDF 10 KB)

Additional file 11: **Top 10 blastn sequence hits for Ma69 MAT genes.** (PDF 430 KB)

Additional file 12: **A list of 16 known amino-acid motifs involved in plant-pathogenicity in other species were used to match the whole proteome datasets of** ***M. anisopliae, M. robertsii*** **and** ***M. acridum*** **via EMBOSS (Preg).** (PDF 78 KB)

Additional file 13: **1620 Ma69 proteins found with motifs linked with pathogenicity in other species.** (XLSX 79 KB)

Additional file 14: **Seven known motifs linked with pathogenicity in other species were matched to the three** ***Metarhizium*** **species investigated.** (PDF 71 KB)

Additional file 15: **1295 putative proteins in Ma69 found with signal peptides.** (XLSX 101 KB)

Additional file 16: **242 candidate effector secreted proteins found in Ma69.** (XLSX 47 KB)

Additional file 17: **Whole genome synteny coordinates from** ***M. anisopliae, M. robertsii,*** **and** ***M. acridum.*** (XLS 5 MB)

Additional file 18: **Whole genome synteny figure comparing the distribution of scaffold length from all three** ***Metarhizium*** **assemblies.** (PNG 28 KB)
