# Supplementary material for: The genome sequence of the biocontrol fungus Metarhizium anisopliae and comparative genomics of Metarhizium species
Source: BMC Genomics. 2014 Aug 7;15(1):660. doi: 10.1186/1471-2164-15-660 (PMC4133081; doi:10.1186/1471-2164-15-660)
Supplement: Supplementary file 1 — Additional file 1: Top ten BLAST hits for Ma69 (complete sequence ITS1; 5.8S rRNA gene; ITS2). (PDF 11 KB) [file 12864_2013_6347_MOESM1_ESM.pdf]

Supplementary Info 1. Top ten BLAST hits for Ma69 (complete sequence ITS1; 5.8S rRNA gene; ITS2)

Id	Description	E-value	Score
316992162	*M. anisopliae* strain ARSEF 7450	0.00	428
316992144	*M. anisopliae* strain ARSEF 7487	0.00	428
345846891	*M. anisopliae* isolate Ma130	0.00	428
345846890	*M. anisopliae* isolate Ma129	0.00	428
344221882	*M. anisopliae* isolate 090919-16-1	0.00	428
254575842	*M. anisopliae* isolate LSVT2	0.00	428
254575817	*M. anisopliae* isolate CNHE	0.00	428
254575813	*M. anisopliae* isolate CNGX	0.00	428
254575804	*M. anisopliae* isolate CNGD6	0.00	428
254575801	*M. anisopliae* isolate CNGD3	0.00	428
