# Supplementary material for: The genome sequence of the biocontrol fungus Metarhizium anisopliae and comparative genomics of Metarhizium species
Source: BMC Genomics. 2014 Aug 7;15(1):660. doi: 10.1186/1471-2164-15-660 (PMC4133081; doi:10.1186/1471-2164-15-660)
Supplement: Supplementary file 7 — Additional file 7: Functional classification of divergent gene in M. anisopliae, M. robertsii and M. acridum : Gene annotations listed for which amino acid translations (predicted proteins) were found by reciprocal-best hit analysis via Proteinortho between the three species. (PDF 51 KB) [file 12864_2013_6347_MOESM7_ESM.pdf]

Supplementary Info 7. Functional classification of divergent genes in *M. anisopliae, M. robertsii* and *M. acridum*. Proteinortho analysis used reciprocal best blast hit relationships to predict orthology between proteins of each *Metarhizium* species. Where a protein lacked orthologs in the other 2 species, we predict these to be 'species-specific-by-orthology' (or divergent).

Function	*M. anisopliae*	*M. robertsii*	*M. acridum*
DNA/RNA binding	13	26	7
Degradative enzymes	6	10	6
Membrane anchored proteins	12	2	3
Transposable elements	14	5	0
Other	44	30	49
Unknown	38	6	22
Total	127	79	87
