# Supplementary material for: The genome sequence of the biocontrol fungus Metarhizium anisopliae and comparative genomics of Metarhizium species
Source: BMC Genomics. 2014 Aug 7;15(1):660. doi: 10.1186/1471-2164-15-660 (PMC4133081; doi:10.1186/1471-2164-15-660)
Supplement: Supplementary file 10 — Additional file 10: BLASTp identification of putative MAT genes from M. anisopliae isolate Ma69. (PDF 10 KB) [file 12864_2013_6347_MOESM10_ESM.pdf]

Supplementary Info 10. BLASTp identification of putative MAT genes from *M. anisopliae* isolate Ma69.

**MAT gene**	**Ma69 gene**	**BLASTp homology**	**NCBI Accession**	**E-value**
MAT1-1-1	8894_g	mating-type A-1 protein [*Metarhizium* anisopliae now robertsii ARSEF 23]	EFZ01122	0
MAT1-1-2	8895_g	MAT1-1-2 [*Metarhizium* anisopliae]	BAE93597	1.0317E-134
MAT1-1-3	8896_g	MAT1-1-3 [*Metarhizium* anisopliae]	BAE93596	1.61149E-95
MAT1-2	3509_g	HMG box transcription factor [*Metarhizium* acridum CQMa 102]	EFY86728	0
