# Supplementary material for: The genome sequence of the biocontrol fungus Metarhizium anisopliae and comparative genomics of Metarhizium species
Source: BMC Genomics. 2014 Aug 7;15(1):660. doi: 10.1186/1471-2164-15-660 (PMC4133081; doi:10.1186/1471-2164-15-660)
Supplement: Supplementary file 11 — Additional file 11: Top 10 blastn sequence hits for Ma69 MAT genes. (PDF 430 KB) [file 12864_2013_6347_MOESM11_ESM.pdf]

Gene	Query sequence	Hit	Id	Description
MAT switching	MA69_3030	EFZ02423	322710849	mating-type switching protein swi10 [Metarhizium anisopliae ARSEF 23]
	MA69_3030	EFY90629	322698862	hypothetical protein MAC_03407 [Metarhizium acridum CQMa 102]
	MA69_3030	EGX95171	346325574	hypothetical protein CCM_03443 [Cordyceps militaris CM01]
	MA69_3030	CCF41447	380482106	hypothetical protein CH063_11716 [Colletotrichum higginsianum]
	MA69_3030	XP_001906015	171682144	hypothetical protein [Podospora anserina S mat+] >gi|170941031|emb|CAP66681.1| unnamed protein product [Podospora anserina S mat+]
	MA69_3030	XP_003046813	302895865	hypothetical protein NECHADRAFT_66834 [Nectria haematococca mpVI 77-13-4] >gi|256727740|gb|EEU41100.1| hypothetical protein NECHADRAFT_66834 [Nectria haematococca mpVI 77-13-4]
	MA69_3030	EGU81381	342880207	hypothetical protein FOXB_08110 [Fusarium oxysporum Fo5176]
	MA69_3030	EGY20453	346977001	hypothetical protein VDAG_10082 [Verticillium dahliae VdLs.17]
	MA69_3030	EFQ33500	310798607	hypothetical protein GLRG_08779 [Glomerella graminicola M1.001]
	MA69_3030	XP_003650455	367040149	hypothetical protein THITE_2109935 [Thielavia terrestris NRRL 8126] >gi|346997716|gb|AEO64119.1| hypothetical protein THITE_2109935 [Thielavia terrestris NRRL 8126]
MAT switching	MA69_3031	EFZ02423	322710849	mating-type switching protein swi10 [Metarhizium anisopliae ARSEF 23]
	MA69_3031	EFY90630	322698863	mating-type switching protein swi10 [Metarhizium acridum CQMa 102]
	MA69_3031	EGU81382	342880208	hypothetical protein FOXB_08111 [Fusarium oxysporum Fo5176]
	MA69_3031	XP_389435	46134239	hypothetical protein FG09259.1 [Gibberella zeae PH-1]
	MA69_3031	EFQ33499	310798606	DNA repair protein rad10 [Glomerella graminicola M1.001]
	MA69_3031	EHK40544	358391140	hypothetical protein TRIATDRAFT_320874 [Trichoderma atroviride IMI 206040]
	MA69_3031	CCF41448	380482107	DNA repair protein rad10 [Colletotrichum higginsianum]
	MA69_3031	XP_003001931	302408192	mating-type switching protein swi10 [Verticillium albo-atrum VaMs.102] >gi|261359652|gb|EEY22080.1| mating-type switching protein swi10 [Verticillium albo-atrum VaMs.102]
	MA69_3031	EGX95668	346326072	mating-type switching protein swi10 [Cordyceps militaris CM01]
	MA69_3031	EHK21504	358383843	hypothetical protein TRIVIDRAFT_152287 [Trichoderma virens Gv29-8]
MAT 1-1-1	MA69_8894	EFZ01122	322709546	mating-type A-1 protein [Metarhizium anisopliae ARSEF 23]
	MA69_8894	ACN59937	224593237	mating-type A-1 protein [Epichloe festucae]
	MA69_8894	ACR78244	238915463	MAT1-1-1 [Hypocrea jecorina]
	MA69_8894	CAQ42990	207099783	mating type protein [Acremonium chrysogenum]
	MA69_8894	BAE93598	94384838	MAT1-1-1 [Metarhizium anisopliae]
	MA69_8894	XP_003052790	302919099	hypothetical protein NECHADRAFT_17696 [Nectria haematococca mpVI 77-13-4] >gi|256733730|gb|EEU47077.1| hypothetical protein NECHADRAFT_17696 [Nectria haematococca mpVI 77-13-4]
	MA69_8894	BAC67541	113460120	MAT1-1-1 [Isaria tenuipes]
	MA69_8894	CAD59611	38141618	putative mating type protein MAT-1-1 [Colletotrichum musae]
	MA69_8894	EGX90103	346320503	mating-type protein MAT 1-1-1 [Cordyceps militaris CM01]
	MA69_8894	EGX90103	346320503	mating-type protein MAT 1-1-1 [Cordyceps militaris CM01]
MAT 1-1-2	MA69_8895	BAE93597	94384837	MAT1-1-2 [Metarhizium anisopliae]
	MA69_8895	AEI72617	337734382	mating type A-1-2 protein [Epichloe festucae]
	MA69_8895	BAD72611	55785714	MAT1-1-2 [Epichloe typhina]
	MA69_8895	BAD72607	55785709	MAT1-1-2 [Ephelis japonica]
	MA69_8895	BAD72603	55785704	MAT1-1-2 [Claviceps purpurea]
	MA69_8895	BAE93601	94384842	MAT1-1-2 [Tolypocladium inflatum]
	MA69_8895	EGX90104	346320504	mating-type protein MAT 1-1-2 [Cordyceps militaris CM01]
	MA69_8895	ACR78245	238915464	MAT1-1-2 [Hypocrea jecorina]
	MA69_8895	BAC67540	29501195	MAT1-1-2 [Isaria tenuipes] >gi|62857325|dbj|BAD95879.1| MAT1-1-2 [Cordyceps takaomontana]
	MA69_8895	BAD72599	55785699	MAT1-1-2 [Cordyceps militaris]
MAT 1-1-3	MA69_8896	BAE93596	94384836	MAT1-1-3 [Metarhizium anisopliae]
	MA69_8896	EFZ01123	322709547	MAT1-1-3 like protein [Metarhizium anisopliae ARSEF 23]
	MA69_8896	AEI72616	337734380	mating type A-1-3 HMG1/2 [Epichloe festucae]
	MA69_8896	BAD72610	95113630	MAT1-1-3 [Epichloe typhina]
	MA69_8896	BAD72606	95113629	MAT1-1-3 [Ephelis japonica]
	MA69_8896	BAD72602	95113628	MAT1-1-3 [Claviceps purpurea]
	MA69_8896	BAE93600	94384841	MAT1-1-3 [Tolypocladium inflatum]
	MA69_8896	CAQ42991	207099787	mating type protein [Acremonium chrysogenum]
	MA69_8896	ACR78246	238915465	MAT1-1-3 [Hypocrea jecorina]
	MA69_8896	AAG42812	11993612	mating type protein MAT1-1-3 [Gibberella zeae]
MAT 1-2	MA69_3509	EFY86728	322694910	HMG box transcription factor [Metarhizium acridum CQMa 102]
	MA69_3509	EGX93214	346323616	SNF2 family helicase, putative [Cordyceps militaris CM01]
	MA69_3509	XP_003051581	302915541	hypothetical protein NECHADRAFT_106035 [Nectria haematococca mpVI 77-13-4] >gi|256732520|gb|EEU45868.1| hypothetical protein NECHADRAFT_106035 [Nectria haematococca mpVI 77-13-4]
	MA69_3509	EGU84400	342884057	hypothetical protein FOXB_05065 [Fusarium oxysporum Fo5176]
	MA69_3509	XP_385327	46121545	hypothetical protein FG05151.1 [Gibberella zeae PH-1]
	MA69_3509	CCF36618	380489566	HMG box protein [Colletotrichum higginsianum]
	MA69_3509	EFQ33384	310798491	HMG box protein [Glomerella graminicola M1.001]
	MA69_3509	XP_002795160	295669224	HMG-box transcription factor [Paracoccidioides brasiliensis Pb01] >gi|226285094|gb|EEH40660.1| HMG-box transcription factor [Paracoccidioides brasiliensis Pb01]
	MA69_3509	XP_002374195	238485914	HMG box protein, putative [Aspergillus flavus NRRL3357] >gi|220699074|gb|EED55413.1| HMG box protein, putative [Aspergillus flavus NRRL3357]
	MA69_3509	EEH20890	225682606	conserved hypothetical protein [Paracoccidioides brasiliensis Pb03]

Gene	E-value	Score	Bit score	Hit start	Hit end	Hit length	Query start	Query end	Overlap	Identity	%Identity	Positive	%Positive	Gaps	%Gaps
MAT switching	0	1692	656.37	1	409	409	33	441	89.69	396	97	399	98	0	0
	0	1403	545.04	1	401	401	33	441	87.94	319	78	345	84	8	2
	1.9838E-32	311	124.41	201	421	221	201	441	48.46	92	36	128	51	44	17
	1.6308E-24	251	101.29	164	405	242	203	437	53.07	83	34	114	46	15	6
	2.51946E-23	242	97.83	177	395	219	201	441	48.03	85	35	109	44	30	12
	2.65457E-23	241	97.44	140	382	243	201	441	53.29	86	32	119	45	46	17
	3.95541E-22	233	94.36	171	410	240	201	439	52.63	78	31	115	45	27	11
	8.66065E-22	230	93.20	174	400	227	201	441	49.78	81	33	114	46	26	11
	1.61954E-21	223	90.51	3	241	239	201	432	52.41	84	34	116	47	25	10
	1.31269E-19	213	86.66	139	378	240	201	441	52.63	86	34	112	44	31	12
MAT switching	0	1772	687.18	470	839	370	1	370	99.73	360	97	363	98	0	0
	0	1662	644.81	44	415	372	1	369	100.27	336	90	351	94	5	1
	1.2624E-165	1204	468.39	43	385	343	1	369	92.45	254	69	298	81	26	7
	4.4482E-159	1160	451.44	39	384	346	1	368	93.26	257	69	303	81	32	9
	3.7328E-156	1143	444.89	50	404	355	1	370	95.69	238	64	280	75	19	5
	6.5209E-156	1141	444.12	58	398	341	14	370	91.91	240	67	272	76	20	6
	1.2614E-152	1120	436.03	50	412	363	1	370	97.84	245	65	291	77	23	6
	9.2737E-151	1108	431.41	55	414	360	1	370	97.04	223	60	274	73	16	4
	1.1082E-150	1105	430.25	62	388	327	13	368	88.14	231	65	264	74	31	9
	2.5277E-149	1089	424.09	22	283	262	1	264	70.62	205	78	228	86	2	1
MAT 1-1-1	0	1829	709.14	1	365	365	1	349	98.92	346	95	349	96	16	4
	0	1470	570.85	1	368	368	1	368	99.73	269	73	316	86	2	1
	3.2823E-121	909	354.76	1	379	379	1	368	102.71	185	48	258	67	21	5
	2.6778E-101	776	303.52	1	371	371	1	364	100.54	166	43	235	61	37	10
	3.81826E-81	618	242.66	1	116	116	1	116	31.44	114	98	115	99	0	0
	1.38918E-67	537	211.46	3	224	222	65	293	60.16	108	47	148	64	13	6
	2.53626E-60	506	199.52	12	241	230	6	225	62.33	104	45	148	64	16	7
	4.43368E-60	497	196.05	52	341	290	59	339	78.59	118	39	161	53	31	10
	9.62145E-60	502	197.98	12	241	230	6	225	62.33	104	45	152	65	16	7
	3.7637E-09	124	52.37	402	456	55	316	368	14.91	22	40	34	62	2	4
MAT 1-1-2	1.0317E-134	975	380.18	1	206	206	1	216	94.93	193	89	197	91	10	5
	1.18802E-78	604	237.27	1	212	212	1	216	97.70	115	53	156	72	4	2
	5.52988E-73	567	223.02	1	215	215	1	216	99.08	113	52	152	69	7	3
	2.49229E-63	503	198.36	1	216	216	1	216	99.54	102	46	150	68	8	4
	2.5077E-56	459	181.42	1	212	212	1	216	97.70	102	47	142	65	10	5
	5.57947E-47	391	155.22	1	226	226	1	216	104.15	77	34	134	59	10	4
	1.51461E-46	388	154.07	1	216	216	1	216	99.54	75	35	132	61	2	1
	5.32968E-45	382	151.75	1	217	217	1	216	100.00	83	38	129	59	7	3
	2.34498E-43	366	145.59	1	212	212	1	216	97.70	77	35	135	62	6	3
	8.24129E-42	355	141.35	1	204	204	1	216	94.01	73	34	126	58	14	6
MAT 1-1-3	1.61149E-95	704	275.79	12	193	182	1	183	98.91	142	75	152	80	13	7
	5.2084E-93	694	271.94	1	181	181	1	182	98.37	139	74	150	80	13	7
	5.08376E-77	582	228.79	13	193	181	2	183	98.37	111	61	136	75	1	1
	3.18846E-75	570	224.17	13	193	181	2	183	98.37	111	58	140	73	19	10
	3.4016E-74	563	221.48	12	193	182	1	183	98.91	109	59	141	76	5	3
	4.44263E-68	523	206.07	13	193	181	2	183	98.37	105	55	132	69	19	10
	7.59148E-55	435	172.17	14	189	176	2	178	95.65	92	51	118	65	11	6
	1.58291E-39	332	132.49	5	186	182	2	183	98.91	75	40	110	59	12	6
	5.05196E-39	330	131.72	14	191	178	1	183	96.74	77	41	101	54	15	8
	1.82502E-36	311	124.41	6	180	175	3	180	95.11	68	37	102	55	17	9
MAT 1-2	0	3109	1202.19	1	685	685	1	685	99.85	630	92	650	95	0	0
	6.5154E-163	1249	485.72	51	667	617	54	685	89.94	314	48	420	65	49	8
	4.4691E-149	1155	449.51	36	653	618	34	679	90.09	301	46	396	60	50	8
	2.533E-140	1097	427.17	31	663	633	34	679	92.27	284	42	378	56	61	9
	5.0219E-140	1094	426.02	32	653	622	35	679	90.67	282	43	388	59	39	6
	1.0127E-122	979	381.72	50	677	628	54	684	91.55	274	41	379	57	65	10
	8.1592E-118	945	368.62	50	677	628	54	684	91.55	266	40	371	56	65	10
	1.25562E-59	538	211.85	125	711	587	87	680	85.57	206	31	291	44	151	23
	3.52022E-59	534	210.31	133	691	559	87	678	81.49	195	31	300	47	125	20
	1.96703E-58	529	208.38	125	707	583	87	680	84.99	205	31	291	44	155	23
