# Supplementary material for: The genome sequence of the biocontrol fungus Metarhizium anisopliae and comparative genomics of Metarhizium species
Source: BMC Genomics. 2014 Aug 7;15(1):660. doi: 10.1186/1471-2164-15-660 (PMC4133081; doi:10.1186/1471-2164-15-660)
Supplement: Supplementary file 12 — Additional file 12: A list of 16 known amino-acid motifs involved in plant-pathogenicity in other species were used to match the whole proteome datasets of M. anisopliae, M. robertsii and M. acridum via EMBOSS (Preg). (PDF 78 KB) [file 12864_2013_6347_MOESM12_ESM.pdf]

Supplementary Info 12. A list of 16 known motifs involved in fungal pathogenicity were used to match the whole proteome datasets of *M. anisopliae*, *M. robertsii* and *M. acridum* via EMBOSS (Preg).

**Motif**	**Reference**
RXLR(X{5,30})EER	Whisson et al. (2007) Nature 450: 115–118, Dou et al. (2008) Plant Cell 20: 1118–1133, Grouffaud et al. (2008) Microbiology 154: 3743–3751
RXLR	Whisson et al. (2007) Nature 450: 115–118, Dou et al. (2008) Plant Cell 20: 1118–1133, Grouffaud et al. (2008) Microbiology 154: 3743–3751
RFYR	Kale et al (2010) Cell 142(2):284-295
RTLK	Kale et al (2010) Cell 142(2):284-295
RYWT	Kale et al (2010) Cell 142(2):284-295
RMLH	Kale et al (2010) Cell 142(2):284-295
RIYER	Kale et al (2010) Cell 142(2):284-295
CHXC	Kemen et al (2011) PLoS Biol 9(7): e1001094
LXLFLAK	Win et al. (2007) Plant Cell 19: 2349–2369, Haas et al. (2009) Nature 461: 393–398
YXSL[RK]	Levesque et al. (2010) Genome Biology 11:R73
[YFW]XC	Godfrey et al (2010) BMC Genomics 11:317
HVLVXXP	Haas et al. (2009) Nature 461: 393–398
[FL]XLYLALK	Gaulin et al. (2008) PloS One 3: e1723
LXLYLAXR	Chueng et al. (2008) BMC Genomics 9: 542
KECXD	Nicastro et al. (2009) Protein Sci 18: 1786–1791
RQHHKR(X{5,15})HRRHK	Kemen et al. (2005) Mol Plant Microbe Interact 18: 1130–1139
[LI]XAR	Yoshida et al. (2009) Plant Cell 21:1573–1591, Li et al. (2009) Mol Plant Microbe Interact 22: 411–420
[RK]CXXC(X{6,18})H	Yoshida et al. (2009) Plant Cell 21:1573–1591
[RK]VY[LI]R	Ridout et al. (2006) Plant Cell 18: 2402–2414

Note: RXLR and RXLR...dEER have been counted as separate motifs. Parentheses “()” indicate a subset of the pattern. Brackets “[]” indicate multiple possible amino-acids at that position. The letter 'X' indicates any amino-acid can be substituted at that position. Braces “{}” indicate the minimum and maximum allowable range of amino-acids at a given position.
