# Supplementary material for: The genome sequence of the biocontrol fungus Metarhizium anisopliae and comparative genomics of Metarhizium species
Source: BMC Genomics. 2014 Aug 7;15(1):660. doi: 10.1186/1471-2164-15-660 (PMC4133081; doi:10.1186/1471-2164-15-660)
Supplement: Supplementary file 14 — Additional file 14: Seven known motifs linked with pathogenicity in other species were matched to the three Metarhizium species investigated. (PDF 71 KB) [file 12864_2013_6347_MOESM14_ESM.pdf]

Supplementary Info 14. Seven known fungal pathogenicity motifs were matched to the three *Metarhizium* species investigated.

Motif	Reference	M. anisopliae	M. robertsii	M. acridum
[LI]XAR	[1]	22	32	21
[RK]CXX(X{6,18})H	[1]	1	4	0
[RK]VY[LI]R	[2]	1	0	0
RXLR	[3]	15	22	17
[YFW]XC	[4]	40	35	26
YXSL[RK]	[5]	0	0	2
RXLR(X{5,30})EER	[3]	0	1	0

Note: Parentheses "( )" indicate a subset of the pattern. Brackets "[ ]" indicate multiple possible amino-acids at that position. The letter 'X' indicates any amino-acid can be substituted at that position. Braces "{ }" indicate the minimum and maximum allowable range of amino-acids at a given position.

1. Yoshida K, Saitoh H, Fujisawa S, Kanzaki H, Matsumura H, Yoshida K, Tosa Y, Chuma I, Takano Y, Win J *et al*: **Association genetics reveals three novel avirulence genes from the rice blast fungal pathogen *Magnaporthe oryzae***. *The Plant Cell* 2009, **21**:1573-1591.
2. Christopher JR, Pari S, Oliver P, Soledad S, Jonathan DGJ, James KMB: **Multiple avirulence paralogues in cereal powdery mildew fungi may contribute to parasite fitness and defeat of plant resistance**. *The Plant Cell* 2006, **18**(9):2402-2414.
3. Whisson SC, Boevink PC, Moleleki L, Avrova AO, Morales JG, Gilroy EM, Armstrong MR, Grouffaud S, van West P, Chapman S *et al*: **A translocation signal for delivery of oomycete effector proteins into host plant cells**. *Nature* 2007, **450**:115-119.
4. Godfrey D, Bohlenius H, Pedersen C, Zhang Z, Emmersen J, Thordal-Christensen H: **Powdery mildew fungal effector candidates share N-terminal Y/F/WxC motif**. *BMC Genomics* 2010, **11**(1):317.
5. Levesque CA, Brouwer H, Cano L, Hamilton J, Holt C, Huitema E, Raffaele S, Robideau G, Thines M, Win J *et al*: **Genome sequence of the necrotrophic plant pathogen Pythium ultimum reveals original pathogenicity mechanisms and effector repertoire**. *Genome Biology* 2010, **11**(7):R73.
