# Supplementary figures and images for: The genome sequence of the biocontrol fungus Metarhizium anisopliae and comparative genomics of Metarhizium species
Source: BMC Genomics. 2014 Aug 7;15(1):660. doi: 10.1186/1471-2164-15-660 (PMC4133081; doi:10.1186/1471-2164-15-660)

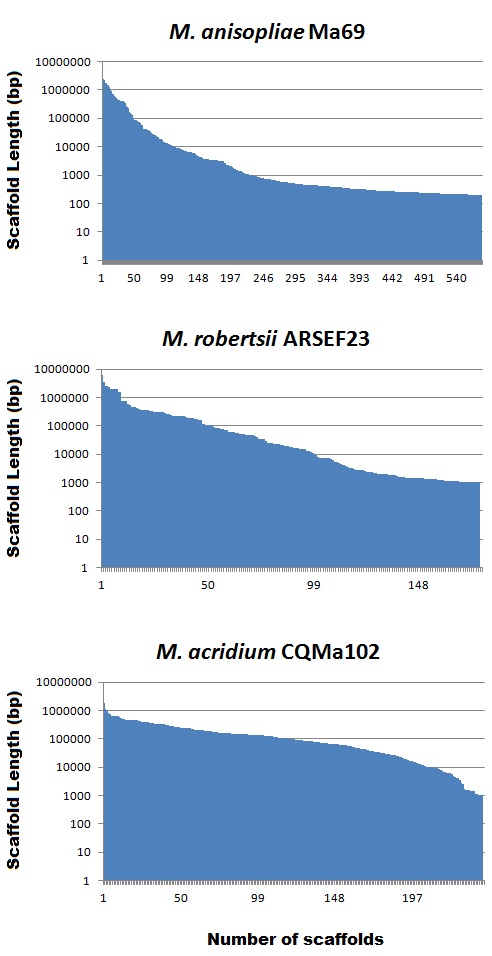

Supplement: Supplementary file 18 — Additional file 18: Whole genome synteny figure comparing the distribution of scaffold length from all three Metarhizium assemblies. (PNG 28 KB) [file 12864_2013_6347_MOESM18_ESM.png]
